# Remarkable Progress with Small-Molecule Modulation of TRPC1/4/5 Channels: Implications for Understanding the Channels in Health and Disease

**DOI:** 10.3390/cells7060052

**Published:** 2018-06-01

**Authors:** Aisling Minard, Claudia C. Bauer, David J. Wright, Hussein N. Rubaiy, Katsuhiko Muraki, David J. Beech, Robin S. Bon

**Affiliations:** 1 School of Chemistry, University of Leeds, Leeds LS2 9JT, UK; cm10a3m@leeds.ac.uk; 2 Department of Discovery and Translational Science, Leeds Institute of Cardiovascular and Metabolic Medicine, University of Leeds, Leeds LS2 9JT, UK; c.bauer@leeds.ac.uk (C.C.B.); d.j.wright1@leeds.ac.uk (D.J.W.); d.j.beech@leeds.ac.uk (D.J.B.); 3 Centre for Atherothrombosis and Metabolic Disease, Hull York Medical School, Hull HU6 7RX, UK; h.rubaiy@hull.ac.uk; 4 Laboratory of Cellular Pharmacology, School of Pharmacy, Aichi-Gakuin University, 1-100 Kusumoto, Chikusa, Nagoya 464-8650, Japan; kmuraki@dpc.agu.ac.jp

**Keywords:** ion channel, TRPC, small molecules, calcium, chemical probes

## Abstract

Proteins of the TRPC family can form many homo- and heterotetrameric cation channels permeable to Na^+^, K^+^ and Ca^2+^. In this review, we focus on channels formed by the isoforms TRPC1, TRPC4 and TRPC5. We review evidence for the formation of different TRPC1/4/5 tetramers, give an overview of recently developed small-molecule TRPC1/4/5 activators and inhibitors, highlight examples of biological roles of TRPC1/4/5 channels in different tissues and pathologies, and discuss how high-quality chemical probes of TRPC1/4/5 modulators can be used to understand the involvement of TRPC1/4/5 channels in physiological and pathophysiological processes.

## 1. Introduction

**Figure 1:**
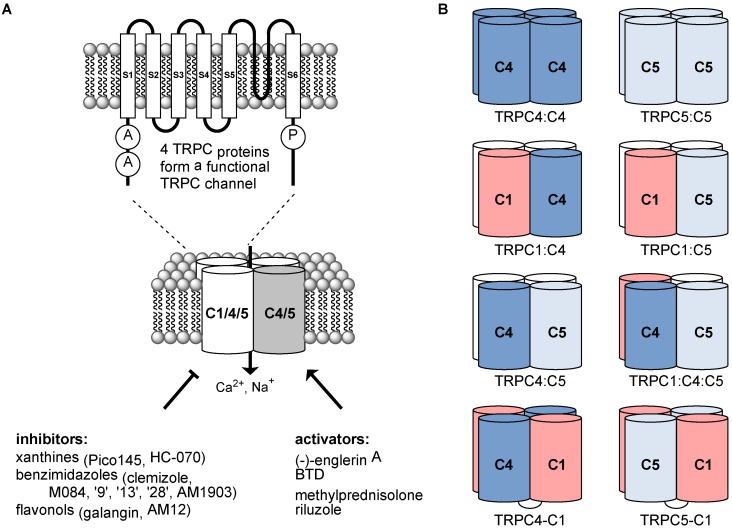
(**A**) Formation of tetrameric TRPC1/4/5 cation channels by TRPC1/4/5 proteins and recently discovered small-molecule modulators discussed in this review. Domains “A” are the ankyrin repeat domains present in all TRPC proteins, and “P” is the PDZ binding domain present in TRPC4 and TRPC5. (**B**) Composition of different TRPC1/4/5 channels discussed in this review. Native channels are believed to exist predominantly as heteromers, but their exact compositions and stoichiometries are often unknown (as depicted by white subunits, which may be TRPC1/4/5 proteins or other TRP(C) proteins). Linked subunits depict recombinant TRPC4–C1 and TRPC5–C1 concatemeric proteins that can be used to control channel stoichiometry.

Transient Receptor Potential (TRP) proteins form tetrameric, non-selective ion channels permeable to Na^+^, K^+^ and—in most instances—Ca^2+^ [1,2,3,4]. The 28 mammalian TRP homologues are divided into six subclasses (TRPM, TRPV, TRPA, TRPP, TRPML and TRPC) according to distinctions at the sequence level [3,5], while TRPN (NOMPC) proteins (present in, for example, fruit flies, nematodes and zebrafish) have no mammalian homologues [3]. Four monomers are needed to form a functional ion channel; more than 28 different mammalian TRP channels can form because channels may consist of homomers or heteromers of subunits (Figure 1), each with their own characteristics and functions. Although there is differential expression of TRPs in different cells, tissues, and in different pathologies, most TRP proteins are broadly expressed in both excitable and non-excitable cells, where they enable coupling of relatively slow chemical and physical events to cellular signalling, either directly or indirectly [1,6,7]. The regulation of some TRP channels by many modulators has led to the idea that the channels are complex integrators of multiple chemical and physical factors [1,8].

There are seven members of the TRPC subfamily, of which TRPC2 is not expressed in humans and the great apes because it is encoded by a pseudogene in these species [9]. TRPC proteins are especially prone to formation of heterotetrameric channels within subgroups (Figure 1), one consisting of TRPC3, TRPC6 and TRPC7 and the other of TRPC1, TRPC4 and TRPC5 [5]. TRPC4 and TRPC5 are the most closely related TRPC proteins [10], with 69% sequence identity (BLAST search [11]). TRPC1, which can interact with proteins from other TRP channel families [12,13], may not form functional homomeric channels, but is an important contributor to heteromeric channels with TRPC4 and/or TRPC5 [1,14,15,16,17,18].

This review focuses on channels composed of TRPC1, TRPC4 and TRPC5 (Section 2), their small-molecule modulators (Section 3) and their roles in health and disease (Section 4), to highlight examples and opportunities for the use of small-molecule TRPC1/4/5 modulators to study the role of these channels in physiology and disease. We have used the following notations (Figure 1B): TRPC1, TRPC4 and TRPC5 (etc.) denote the different proteins or any channels incorporating them; TRPC1/4/5 denotes channels composed of TRPC1, TRPC4 and/or TRPC5 (homo- or heteromeric; any ratio); TRPC4:C4 and TRPC5:C5 (etc.) denote specific homomeric channels; TRPC1:C5, TRPC1:C4, TRPC4:C5 and TRPC1:C4:C5 denote specific heteromeric channels (any ratio); and TRPC4–C1 and TRPC5–C1 denote (channels composed of) recombinant, concatemeric proteins (fusions of TRPC1 at the C-terminus of either TRPC4 or TRPC5 through a short linker) that have been developed in our lab to control channel stoichiometry [15,19,20]. The majority of TRPC1/4/5 channels discussed in this review are human and rodent homologues, and where relevant to the discussion, species have been annotated.

## 2. Composition of TRPC1/4/5 Tetrameric Channels

The tetrameric nature of TRPC channels was originally predicted from their homology to voltage-gated potassium channels such as K_V_1.2, for which a crystal structure is available [21]. Current data suggest that TRPC proteins form a variety of homo- and heterotetrameric channels (see below). The recent “resolution revolution” in cryo-electron microscopy [22] has led to the determination of a wide variety of high resolution ion channel structures, providing novel insights into ion channel function. There are examples of TRP channel high resolution structures from each TRP subfamily, which confirm their tetrameric structures [23,24,25,26,27]. Recently, cryo-EM structures of of several TRPC channels, including human TRPC3:C3 [28], human TRPC6:C6 [29], mouse TRPC4:C4 [30] and zebrafish TRPC4:C4 [31] have been reported. hTRPC3 and hTRPC6 share limited homology with hTRPC1, hTRPC4 and hTRPC5 proteins (approximately 40% sequence identity). It is noteworthy that the hTRPC6/C6 channel structure was determined in the presence of a small molecule inhibitor, BTDM, which is bound at a similar position to resiniferatoxin and capsaicin in TRPV1 structures [32,33], between the pore-forming region of one subunit and the voltage sensing-like domain (VSLD) of another. mTRPC4 shares 97% sequence identity with hTRPC4, 70% identity with hTRPC5 and 48% identity with hTRPC1 and so mTRPC4:C4 is the closest homologue to hTRPC1/4/5 channels that has been structurally characterised. It is interesting that in both the mouse and zebrafish TRPC4:C4 structures, a disulfide bond is observed between Cys549 and Cys554 (numbered according to the mouse gene); these disulfides have been previously implicated in channel gating in TRPC5 [34,35] and are conserved only in TRPC1, TRPC4 and TRPC5. Both TRPC4:C4 structures were solved in their closed state in the absence of modulators, so more structural information is required to understand the gating mechanisms of these channels. Additionally, there are only three unique heteromeric structures of any ion channels [36,37,38] and none of these contain TRP proteins. Therefore, to probe the heteromerisation of TRPC channels, we must rely on indirect measurements of their interaction. In this section, we describe evidence for the formation of different TRPC1/4/5 channels, in both overexpression systems and as native channels.

### 2.1. Channels Formed by Overexpressed TRPC1/4/5 Proteins

**Figure 2:**
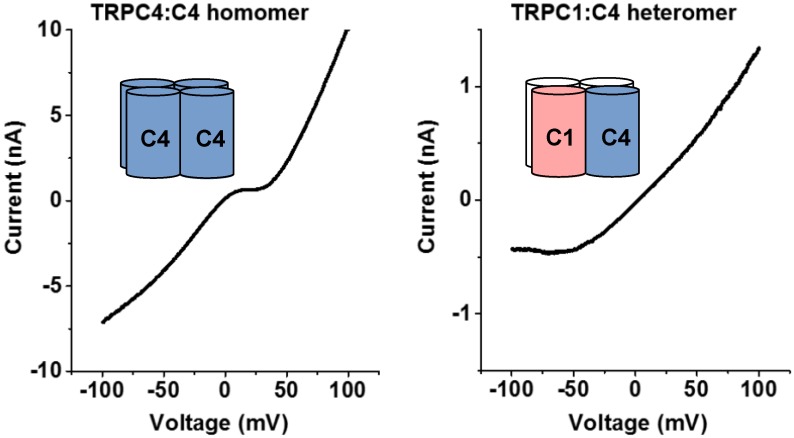
Example I–V plots of homomeric (**left**) and heteromeric/concatemeric (**right**) TRPC1/4/5 channels. Recordings from overexpressed TRPC4:C4 and TRPC1:C4 channels are shown. For examples of native I–V plots, see references [14,16].

There is some evidence to suggest that TRPC1, when overexpressed, is retained in the endoplasmic reticulum (ER) but is found at the plasma membrane when co-expressed with TRPC4 or TRPC5 [39]. Förster Resonance Energy Transfer (FRET) experiments using TRPC1 labelled with Cyan Fluorescent Protein (CFP) and Yellow Fluorescent Protein (YFP) suggest that there are at least two TRPC1 monomers in the tetrameric complex at the ER. The authors suggest the formation of a homotetrameric TRPC1 channel; however, these data do not explicitly rule out a TRPC1 interaction with other natively expressed ion channel monomers, such as Orai1, which has been implicated in the formation of heteromeric channels with TRPC1 [40]. Additionally, it was observed that TRPC4 and TRPC5, when co-expressed with TRPC1, showed different I–V relationships (for examples, see Figure 2), a lower calcium flux, and increased selectivity to sodium ions compared with TRPC4:C4 or TRPC5:C5 homomeric channels [41]. Another study, involving FRET and co-transfected HEK293 cells, demonstrated that TRPC1, TRPC4 and TRPC5 were able to form homomers, that TRPC1 could interact with either TRPC4 or TRPC5, and that TRPC4 and TRPC5 could interact with each other. In this study, co-immunoprecipitation also suggested each homomeric interaction and the same heteromeric interactions between TRPC4 and TRPC5, and TRPC4 and TRPC1 [42]. However, these FRET and co-immunoprecipitation data do not provide direct insight into stoichiometries of heteromeric channels. In addition, stoichiometries may vary depending on expression levels of different TRPC proteins.

Atomic Force Microscopy (AFM) was used to probe TRPC1 tetrameric structure when overexpressed in and purified from HEK293 cells [43]. The authors measured the angles between antibodies binding to TRPC1 (approximately 90 and 180 degrees), which suggested that TRPC1 forms tetramers. Considering TRPC1 was transiently overexpressed, it is surprising that observed tetramers were only seen with two or fewer antibodies bound, which could suggest the formation of heterotetramers with other natively expressed channel proteins from the TRP and other families. The majority of particle sizes were, however, consistent with monomeric TRPC1, suggesting that tetramers were broken up during purification in 3-[(3-cholamidopropyl) dimethylammonio]-1-propanesulfonate (CHAPS) detergent.

We have recently demonstrated that recombinant, concatemeric TRPC4–C1 or TRPC5–C1 proteins can be overexpressed to form functional channels, the I–V relationships and reduced Ca^2+^ permeability of which closely resemble those of native heteromeric TRPC1:C4 and TRPC1:C5 channels [15,19,20]. These concatemers allow the functional analysis of TRPC heterotetramers with fixed stoichiometry, which will be critical to the development of small molecules specific to homo- or heterotetramers [20].

### 2.2. Native TRPC1/4/5 Channels

It has been observed that TRPC proteins show differential tissue expression [44]. Since TRPC1 is expressed in a wide variety of tissues and native I–V plots [14,16] resemble overexpressed heteromeric or concatemeric channels (Figure 2), it is likely that TRPC1 is predominantly observed in heterotetrameric channels in vivo. Additionally, there are several examples of detecting TRPC heterotetramerisation ex vivo that largely agree with the above overexpression studies. For example, TRPC1, TRPC4 or TRPC5 were purified from mouse hippocampal cells using antibodies specific to any one isoform [18]. These data suggest the formation of a tetramer containing TRPC1, TRPC4 and TRPC5; however, alternatively there could be populations of TRPC1/4, TRPC1/5 and TRPC4/5 channels, which would result in similar co-immunoprecipitation results. In a second example, TRPC4 and TRPC5, after formaldehyde crosslinking, were co-immunoprecipitated from bovine aortic endothelial cells [45]. These results suggest that different tetrameric TRPC1/4/5 channels are formed in vivo.

Further complicating the field of TRPC heterotetramerisation is the existence of splice isoforms of TRPC proteins; two have been reported for TRPC1 [46,47], and seven for TRPC4 [48,49]. Of the TRPC4 isoforms, TRPC4α and TRPC4β have been studied in most detail. TRPC4α and TRPC4β (which lacks 84 residues towards the C terminus) show differential tissue expression. TRPC4β:C4β channels no longer respond to phosphatatidylinositol 4,5-bisphosphate [50], but are still activated by (−)-englerin A [51]. These splice variants are likely to result in even more heterogeneity in TRPC1/4/5 channels.

In summary, there has been much progress in the field of TRPC1/4/5 heterotetramer identification. It should be noted that interactions found in co-immunoprecipitation experiments with detergent-solubilised TRPC1/4/5 proteins may not accurately reflect interactions in native membranes, and even in experiments that involve formaldehyde crosslinking before cell lysis, it may be difficult to exclude interactions between different homomeric channels during co-immunoprecipitations. However, the combination of co-immunoprecipitation results and the fact that I–V plots of native channels closely resemble those of cells that either co-express TRPC4/5 and TRPC1, or express TRPC4–C1 or TRPC5–C1 concatemers, strongly suggests that TRPC1, TRPC4 and TRPC5 form functional heterotetrameric channels in different tissues. There is currently no firm evidence to suggest the native stoichiometry—or, more probably, stoichiometries—of these multimers though.

## 3. Recent Progress with Small-Molecule Modulators of TRPC1/4/5 Channels

TRPC1/4/5 channels are modulated by a wide range of physiological factors, and physical and chemical stimuli, including temperature, redox status, G-protein signalling, endogenous lipids, heavy metal ions, dietary lipids, natural products and synthetic small molecules. We and others have previously reviewed the development of small-molecule modulators of TRP(C) channels [1,52]. Traditionally, TRPC1/4/5 channels have often been activated with lanthanide ions (La^3+^, Gd^3+^), GPCR agonists such as carbachol, or small molecules such as rosiglitazone (which is non-specific and has low potency), and inhibited with the non-specific small molecules such as 2-APB or SFK96365. In addition, for most traditional small-molecule TRPC1/4/5 modulators, little is known about their mode-of-action, and cellular targets of these molecules are likely diverse. Although some TRPC1/4/5 modulators are thought to bind directly to the channels, no small-molecule binding sites have been identified so far.

**Figure 3:**
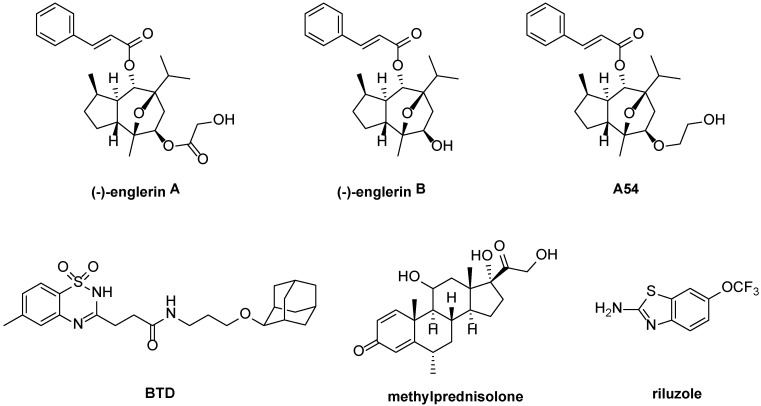
Structures of recently reported TRPC1/4/5 activators, the (−)EA metabolite (−)EB, and the (−)EA antagonist A54.

In 2011, Miller et al. reported ML204 (Figure 3) as a low micromolar inhibitor of TRPC4 and TRPC5 channels that did not inhibit other TRP channels and lacked binding to a panel of 68 receptors [53], leading to use in several studies of the roles of TRPC1/4/5 channels (see Section 4). However, recent studies suggest that ML204 is a relatively poor inhibitor of TRPC1:C4 and TRPC1:C5 channels, at least when channels are activated by (−)-englerin A [16,19]. Because most native TRPC1/4/5 channels are thought to be heteromeric, this needs to be taken into account when using ML204 for functional studies.

Since 2013, remarkable progress has been made with the discovery and development of potent and efficacious small-molecule modulators with unique selectivity profiles and improved pharmacological properties. In this section, we highlight selected small-molecule TRPC1/4/5 modulators reported since our previous review of the field [1].

### 3.1. Activators

#### 3.1.1. Englerins

Screening of organic extracts from the East African plant *Phyllanthus engleri* against the NCI 60 cancer cell panel, followed by bioactivity-guided fractionation, led to the identification of the sequiterpene natural product (−)-englerin A ((−)EA; Figure 3) as a compound with highly selective cytotoxicity against renal carcinoma cell lines [54,55]. Independent target identification approaches by the groups of Waldmann, Christmann and Beech [14,15] and by Novartis [51] revealed that (−)EA is a potent and efficacious activator (EC_50_ = 10 nM) of native TRPC1:C4 channels in A498 renal cancer cells (see Section 4.3 for more detail about its relevance to cancer cell death).

Subsequent experiments revealed that (−)EA activates TRPC4:C4 and TRPC5:C5 channels with low nanomolar EC_50_ values (11 and 7 nM, respectively) and a strong stimulatory effect on both intracellular Ca^2+^ levels and TRPC4:C4 and TRPC5:C5 ionic currents [14]. (−)EA has similar activating effects on heteromeric TRPC1:C4 and TRPC1:C5 channels, but TRPC6, TRPM2 and TRPV4 channels, 10 other ion channels, and 59 GPCRs lack responses to (−)EA [14,51]. (−)EA has been proposed to affect protein kinase C isoform θ (PKCθ) [56] and L-type calcium channels as well [57], although at higher concentrations (most experiments were done with 1–10 µM of (−)EA). Despite extensive target identification campaigns, no further targets have been found [14,51]. This suggests that (−)EA is a highly selective activator of TRPC1/4/5 channels.

The molecular mechanism by which (−)EA selectively activates TRPC1/4/5 channels is not understood. Excised membrane patch recordings in the presence or absence of G protein blockade suggest that (−)EA activates TRPC4/5 channels directly via a site exposed extracellularly or accessible only via the external leaflet of the bilayer [12]. The recent identification of A54 (Figure 3), a competitive antagonist of (−)EA-induced (but not Gd^3+^-induced) TRPC4/5 activation, suggests the presence of a well-defined (−)EA binding site in TRPC4/5 channels [58]. Carson et al. found (−)EA to be stable in human and canine plasma. However, in plasma from rats and mice, (−)EA converts to the inactive metabolite (−)-englerin B ((−)EB; resulting from glycolate ester hydrolysis; Figure 3) [51]. These effects were recapitulated in vivo upon oral dosing of 5 mg/kg in rodents: (−)EA blood levels did not rise above 12 nM, but (−)EB levels of >50 nM were detected. (−)EB neither activates TRPC1/4/5 channels nor is a potent A498 killer and also glycolic acid is inactive [51,59]. (−)EA is acutely toxic to rodents, although higher doses are tolerated upon intraperitoneal or subcutaneous injection than upon intravenous administration and toxicity may depend on drug formulation [51,60]. In contrast, (−)EB does not show toxicity to rodents [51].

#### 3.1.2. BTD and Methylprednisolone

Through a screen of a ChemBioNet compound library against (mouse) TRPC5, Beckmann et al. found two novel TRPC5 activators: the glucocorticoid methylprednisolone (EC_50_ = 12 µM) and *N*-[3-(adamantan-2-yloxy)propyl]-3-(6-methyl-1,1-dioxo-2*H*-1λ^6^,2,4-benzothiadiazin-3-yl)propanamide (BTD; EC_50_ = 1.4 µM) (Figure 3) [61]. The TRPC5 activation by these compounds is long-lasting, reversible, and sensitive to the recently published TRPC5 inhibitor clemizole (see Section 3.2.2). The more potent compound in this study, BTD, was studied in most detail. Although far less potent than (−)EA, BTD has remarkable selectivity on TRPC1/4/5 channel subtypes: patch clamp recordings revealed that BTD activates homomeric TRPC5:C5 channels as well as heteromeric TRPC1:C5 and (putative) TRPC4:C5 channels, but not TRPC4:C4 and TRPC1:C4 channels. The fact that BTD does not affect phospholipase C signalling, and activates TRPC5:C5 channels in inside-out excised membrane patches when applied from the intracellular side, suggests that the compound has a direct effect on TRPC5 channels. BTD has no effect on channels formed by TRPC3, TRPC6, TRPC7, TRPA1, TRPV1, TRPV2, TRPV3, TRPV4, TRPM2 and TRPM3. Methylprednisolone also showed selectivity for TRPC5 channels over other TRP channels, but can potentiate carbachol-induced TRPC4 activation.

#### 3.1.3. Riluzole

Riluzole (Figure 3) is a marketed drug that delays the progression of amyotrophic lateral sclerosis (ALS) [62], and it also has anti-depressant properties [63]. Its wide-ranging effects on neural activity—in particular the neuromotor system—are thought to result from its effect on multiple ion channels; a review of the neural mechanisms of action of riluzole in ALS has been published by Bellingham [64]. Through a medium-throughput screen on mTRPC5-expressing HEK293 cells, Richter et al. found that riluzole activates TRPC5 channels with an EC_50_ of 9.2 μM [65]. Riluzole also activates overexpressed heteromeric TRPC1:C5 channels and endogenous TRPC5 channels in the U-87 glioblastoma cell line. The riluzole-induced TRPC5 activation is mechanistically different from La^3+^-mediated activation. TRPC5 activation by riluzole is reversible upon washout, independent of G protein signalling and PLC activity, and occurs in both inside-out and cell-attached patches. These data suggest a relatively direct mechanism of action on TRPC5 channels.

### 3.2. Inhibitors

#### 3.2.1. Xanthines

**Figure 4:**
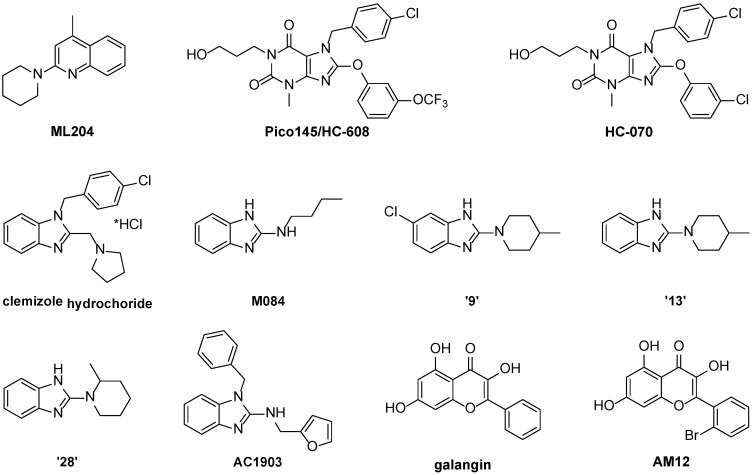
Structures of ML204 and recently reported TRPC1/4/5 inhibitors.

A patent by Hydra Biosciences and Boehringer-Ingelheim claims substituted xanthines and their use as TRPC5 inhibitors [66]. Circa 20% of the 621 compounds therein were reported to have IC_50_ values <100 nM, and eight compounds were further tested in rodent models of anxiety/depression. Studies from two different groups on the effects of the two most promising compounds in the patent have now been published. Our lab reported Pico145 (later called HC-608 by its inventors; Figure 4) as the most potent inhibitor of TRPC1/4/5 channels known to date [19,20]. In calcium recordings, Pico145 inhibits TRPC4 and TRPC5 with IC_50_ values of 349 pM and 1.3 nM, respectively. However, the highest potencies were measured against heteromeric channels (formed by TRPC4–C1 or TRPC5–C1 concatemers; IC_50_ values of 33 pM and 199 pM, respectively) and (−)EA-activated, endogenous TRPC1:C4 channels in A498 renal carcinoma cells (IC_50_ = 49 pM). In whole-cell recordings, Pico145 inhibits both inward and outward currents of TRPC4–C1 with picomolar IC_50_ values, upon activation with either (−)EA or the physiological TRPC4/C5 agonist sphingosine-1-phosphate (S1P). In contrast, 100 nM Pico145 does not affect activities of TRPC3, TRPC6, TRPV1, TRPV4, TRPA1, TRPM2, TRPM8 or store-operated Ca^2+^ entry mediated by Orai1.

The molecular mechanism by which Pico145 selectively inhibits TRPC1/4/5 channels—and distinguishes between specific tetramers—is not understood. Excised outside-out membrane patch recordings suggest that Pico145 inhibits TRPC4 channels directly via a site exposed extracellularly or accessible only via the external leaflet of the bilayer, in a manner independent of cellular signalling mechanisms or Ca^2+^ concentrations, and that the potency of Pico145 depends partially on the concentration of the agonist (−)EA [19]. In addition, the rather mild voltage-dependence of the block does not support the idea of blockage deep inside the ion pore and electric field. Pico145 can also inhibit TRPC4 channels activated with the direct agonist Gd^3+^, although at low concentrations (10 pM), Pico145 can also potentiate Gd^3+^-induced currents mediated by TRPC4 [19]. These data, in combination with the ability of Pico145 to distinguish between closely related channels, suggest that Pico145 occupies a well-defined binding site essential to TRPC4/5 channel gating.

Recently, Just et al. reported the anxiolytic and antidepressant effects in mice of HC-070 (Figure 4), a close analogue of Pico145/HC-608 (for details, see Section 4.1.1) [67]. As part of this study, the activities of HC-070 were tested against TRPC1/4/5 channels (including human, mouse and rat versions) activated by La^3+^ or carbachol (the latter in combination with overexpression of muscarinic receptors), giving IC_50_ values between 0.3 and 2 nM. In addition, both Pico145/HC-608 and HC-070 were subjected to substantial selectivity profiling against a large set of ion channels, receptors, enzymes, kinases and transporters. At 1–2 µM (their solubility limit in Ringer’s buffer), both compounds showed less than 50% inhibition of almost all tested targets. In addition, HC-070 [67] and Pico145 [66] have suitable pharmacokinetic properties for oral dosing. The excellent potency and selectivity of Pico145 and HC-070, in combination with their pharmacokinetic profiles and ready availability—both compounds can be synthesised in three steps from commercially available precursors—make these compounds highly suitable for functional studies of TRPC1/4/5 channels in cells and animals. These data also suggest that small molecules can be pharmacologically distinctive (by almost 40-fold for Pico145) for specific members of the TRPC1/4/5 subfamily, and that the development of multimer-specific inhibitors may be feasible.

#### 3.2.2. Benzimidazoles

Several derivatives of benzimidazole and 2-aminobenzimidazole have been reported as TRPC1/4/5 inhibitors. Clemizole hydrochloride (Figure 4) was originally developed as a histamine H_1_-receptor agonist [68]. However Richter et al. identified it as a novel inhibitor of (mouse) TRPC5:C5 channels with an IC_50_ of 1.1 µM [69]. Clemizole inhibits TRPC5:C5 channels reversibly and inhibition is irrespective of activation mode. TRPC4β:C4β channels are also inhibited by clemizole with an IC_50_ of 6.4 µM. In whole-cell patch-clamp recordings, 10 µM clemizole inhibits heteromeric TRPC1:C5 channels, and 50 µM clemizole partially inhibits riluzole-activated currents in the U-87 glioblastoma cell line. However, clemizole has limited selectivity, as it also inhibits channels formed by TRPC3 (IC_50_ = 9.1 µM), TRPC6 (IC_50_ = 11.3 µM) and TRPC7 (IC_50_ = 26.5 µM).

Following a high-throughput screen of 305,000 compounds (the same campaign that afforded the TRPC4/5 inhibitor ML204), M084 (Figure 4) was identified as a reversible inhibitor of (mouse) TRPC4 and TRPC5 with better stability and inhibitory kinetics than ML204 [70]. Because of relatively low potency of M084 against TRPC4:C4 (IC_50_ = 3.7–10.3 µM), TRPC5:C5 (IC_50_ = 8.2 µM) and TRPC1:C4 (IC_50_ = 8.3 µM), and its slight inhibition of TRPC3 (IC_50_ ~ 50 µM) and TRPC6 (IC_50_ ~ 60 µM), a series of 28 further 2-aminobenzimidazoles was generated and tested, suggesting that 2-aminobenzimidazoles and 2-aminoquinolines (such as ML204) have similar structure-activity relationship (SAR) profiles, and leading to three compounds (“9”, “13” and “28”; Figure 4) with slightly higher potency than M084 (IC_50_ values between 3.1 and 6.6 µM) that do not inhibit TRPC3 and TRPC6. At 30 µM, M084 and its analogues “9”, “13” and “28” do not activate or inhibit Ca^2+^ influx mediated by TRPA1, TRPM8, TRPV1 or TRPV3. The compounds inhibit TRPC4-mediated currents when applied from the extracellular side, and inhibition is not dependent on activation mode. In addition, at 30–100 µM, these compounds block the plateau potential mediated by TRPC4-containing channels in mouse lateral septal neurons.

A recent report on the use of small-molecule TRPC5 inhibitors to suppress progressive kidney disease (see Section 4.2) included the identification of AC1903 (Figure 4) as a selective TRPC5 inhibitor [71]. AC1903 shares structural similarities with both clemizole and M084 (Figure 4), and is equipotent to ML204 against riluzole-evoked TRPC5-mediated currents in whole-cell patch recordings (IC_50_ values of 13.6 and 14.7 µM, respectively). AC1903 is a weak inhibitor of TRPC4 (IC_50_ > 100 µM) and does not inhibit TRPC6 (no inhibition at 100 µM). In standard kinase profiling assays, AC1903 did not show off-target effects. No further selectivity assays on ion channels and receptors were reported, and it is not clear what the effect of AC1903 is on heteromeric TRPC1/4/5 channels.

#### 3.2.3. Flavonols

Several dietary factors, including lipids and polyphenols, are known to inhibit TRPC channels [1]. More recently, we reported the identification of galangin (Figure 4), a natural product from *Alpinia officinarum* and other members of the ginger family, as a TRPC5 inhibitor [72]. Galangin inhibits homomeric TRPC5:C5 with an IC_50_ of 0.45 µM. In addition, galangin inhibits the basal (IC_50_ = 1.9 µM) and La^3+^-evoked (IC_50_ = 6.1 µM) Ca^2+^ responses of differentiated 3T3-L1 cells (a model of mature adipocytes), which are thought to be mediated by heteromeric TRPC1:C5 channels. Subsequent structure-activity relationship (SAR) studies of 48 natural and synthetic flavonols led to the discovery of the more potent analogue AM12 (Figure 4), which inhibits TRPC5:C5 with an IC_50_ of 0.28 µM but is a relatively weak inhibitor of TRPC1:C5 channels. AM12 has no significant inhibitory effect on TRPC3, TRPV4, TRPM2 and store-operated Ca^2+^ release. The reversible inhibition by AM12 of (−)EA-evoked currents of TRPC4:C4 and TRPC5:C5 in outside-out excised membrane patches suggest a relatively direct effect on the channels. However, the effect of AM12 is dependent on the mode of activation; AM12 potentiates TRPC5 when stimulated with S1P or lysophosphatidylcholine (LPC) rather than (−)EA or Gd^3+^. The SAR of the flavonol series also revealed that subtle changes to the flavonol structure can have major impacts on TRPC5 modulatory activity.

### 3.3. Choosing TRPC1/4/5 Modulators for Studies in Cells, Tissues and Animals

The effects of selected small-molecule TRPC1/4/5 modulators have been summarised in Table 1 and Table 2. These compounds (and others described in this review) were profiled by different research groups using a variety of assays (e.g., fluorometric Ca^2+^ and Tl^+^ measurements, calcium imaging, whole-cell patch recordings, excised membrane patch recordings, single channel recordings) in a variety of cell lines, and against TRPC1/4/5 channels from different species (usually the closely related human or mouse homologues). In addition, in inhibition assays, a wide range of activation mechanisms was used, including (−)EA, lanthanides, carbachol (often with overexpression of muscarinic receptors), and riluzole. Such differences need to be taken into account during the design of studies in cells or animals that make use of TRPC1/4/5 modulators.

Currently, the most promising TRPC1/4/5 activator for functional studies is (−)EA, which has unrivalled potency, efficacy and selectivity, making it a valuable probe of TRPC1/4/5 in cellular studies. However, its toxicity and instability in rodent serum and in the gastrointestinal (GI) tract limit its use for in vivo studies. BTD has the advantage that it activates (mouse) TRPC5 channels selectively with respect to TRPC4:C4 and TRPC1:C4 channels. It is selective against several other TRP channels, but potential off-targets have not been profiled comprehensively yet. The marketed drug riluzole can be used in vivo, but has relatively low potency and is thought to affect many ion channels [64].

The most promising TRPC1/4/5 inhibitors for functional studies are Pico145/HC-608 and HC-070. These compounds inhibit TRPC1/4/5 channels at (sub)nM concentrations, while at concentrations up to 1–2 µM, no significant effects on many other proteins have been found. Both compounds are orally bioavailable and are suitable for in vivo studies. AC1903 is an interesting compound because it can distinguish between TRPC5:C5 and TRPC4:C4 channels, while having no effect on TRPC6 or on a panel of kinases. In addition, its pharmacokinetic profile is compatible with in vivo use. However, its relatively low potency and unknown effects on different TRPC1/4/5 tetramers limit its current use as a chemical probe.

## 4. Using Small Molecules to Unravel (Patho)physiological Roles of TRPC1/4/5 Channels

Although observational clinical studies and changes detected in genetically- or pharmacologically-modified rodents and/or human tissue suggest multiple physiological roles of TRPC4/5 channels [1,73], disruption of the *Trpc4/5* genes [74] and global expression of a dominant-negative mutant TRPC5 [17] do not cause catastrophic phenotypes. However, TRPC1/4/5 channels have been implicated in various human diseases, including seizures (TRPC5 and TRPC1:C4) [75], fear-related behaviour (TRPC5) [76,77], severe pulmonary arterial hypertension (TRPC4) [78,79,80], heart failure (TRPC1/C4) [81], and chemotherapeutic resistance of cancers (TRPC5) [82,83]. This section contains a selection of recent studies on the roles of TRPC1/4/5 channels in health and disease to highlight examples of, and opportunities for, the use of small-molecule TRPC1/4/5 modulators to unravel TRPC1/4/5-mediated biological processes.

### 4.1. Roles of TRPC1/4/5 Channels in the Central Nervous System and Pain

#### 4.1.1. Anxiety and Depression

One of the most researched areas of the role of TRPC1/4/5 channels is their potential involvement in the treatment of anxiety and depression. Evidence for these roles comes from studies utilising both transgenic mouse models and pharmacological modulators of these channels.

TRPC5 is expressed in brain regions associated with fear and anxiety, and *Trpc5*^−/−^ mice show decreased fear behaviour compared to wild-type mice in behavioural tests [76], which was attributed to reduced potentiation of TRPC5 currents by G_q/11_-coupled receptors, specifically those stimulated by glutamate and cholecystokinin 2 [76]. A similar anxiolytic phenotype was seen in mice lacking the TRPC4 subunit [84]. In addition, this study showed that TRPC4 protein knockdown limited to the lateral amygdala region—a region of the brain implicated in anxiety—showed the same phenotype as global *Trpc4*^−/−^ mice. This suggests that both TRPC4 and TRPC5 channels in this specific area of the brain may be involved in the development of fear behaviours. It is not known if these TRPC4 and TRPC5 channels are homomers or heteromers, and whether TRPC1 is involved as well.

This proposed role of TRPC4/5 channels in fear behaviour has led to TRPC1/4/5 modulators being investigated as a possible treatment for anxiety. Indeed, the TRPC1/4/5 inhibitor M084 (see Section 3.2.2) [85] has anxiolytic and antidepressant effects in mice [77]. However, it is unknown whether this action of ML084 is due to its effects on homomeric or heteromeric channels, and whether the effect is specifically due to inhibition of TRPC4/5 channels located in the amygdala.

The xanthine HC-070, a highly potent and selective inhibitor of both homo- and heteromeric TRPC1/4/5 channels (see Section 3.2.1), reduces currents stimulated by CCK4 in basolateral amygdala in brain slices, and additionally shows anxiolytic and antidepressant effects in mouse behavioural studies, further confirming these channels as promising clinical targets in the treatment of anxiety and depression [67].

#### 4.1.2. Epilepsy

TRPC1/4/5 channels have also been implicated in epilepsy. TRPC5 channels are highly expressed in rat hippocampal CA1 neurons, where they are thought to be involved in the formation of a prolonged depolarisation, the so-called plateau potential, following cholinergic innervation [86]. Thus far, inhibition of this process was only demonstrated with the non-specific compound 2-APB and with intracellular ATP. The effects of newer pharmacological modulators that show increased potency and selectivity are not known. Additionally, TRPC5 and TRPC1:4 channels are thought to be involved in epileptogenesis in mice, but via distinct expression patterns and mechanisms [75].

Evidence from human studies is limited, however both TRPC1 and TRPC4 proteins are upregulated in brain tissues of patients with focal cortical dysplasia, a common cause of refractory epilepsy [87,88]. Additionally, TRPC4 channel variants have also been implicated in generalised epilepsy [89]. Whether neuronal activity can be modulated by specific activators and inhibitors of TRPC1/4/5 remains to be elucidated.

#### 4.1.3. Pain

TRPC1/4/5 channels have also been implicated in different types of pain. Westlund et al. investigated the role of TRPC4 channels in pain, using mice with a global knockout of the *Trpc4* gene [90]. The *Trpc4*^−/−^ mice showed a resistance to mustard-oil induced visceral pain, as well as increased pain thresholds as compared to wild-type mice. In addition, animals treated with ML204 (0.5 and 1 mg/kg; orally administered) displayed reduced pain behaviours, similar to knockout mice. Wei et al. also investigated the role of TRPC4/5 channels in a spared nerve injury model of neuropathic pain with the TRPC4/5 inhibitor ML204 administered directly into the amygdala [91]. Administration of 5–10 µg ML204 decreased pain behaviour and showed an anti-hypersensitivity effect, which was not present when ML204 was injected into a control site.

#### 4.1.4. Memory

Bröker-Lai et al. recently reported the presence of either TRPC1:C4:C5 channels or mixed populations of TRPC1:C4, TRPC1:C5 and TRPC4:C5 channels in mouse hippocampal cells (see Section 2.2) [18]. In this study, neurons and hippocampal slices from *Trpc1/Trpc4/Trpc5* triple knockout mice showed decreased (action potential-triggered) post-synaptic responses, while the animals displayed impaired cross-frequency coupling in hippocampal networks and deficits in spatial working memory and learning/adaptation. To date, the use of small-molecule TRPC1/4/5 modulators in studies of working memory and learning has not been reported, and it would be important to study whether TRPC1/4/5 inhibitors can have adverse effects on memory.

### 4.2. Roles of TRPC1/4/5 Channels in Kidney Disease

A role for TRPC1/4/5 has been postulated in the kidney, specifically in the development of kidney disease, however the literature provides conflicting findings in this field. TRPC5 channels are expressed in kidney podocytes, specialised cells that form the kidney filter. The channels form a molecular complex with the GTPase Rac1, and are involved in the regulation of cell migration and actin remodelling downstream of angiotensin stimulation [92]. *Trpc5* knockout protects mice from kidney filter barrier damage and resultant albuminuria caused by lipopolysaccharide (LPS), as well as protecting podocytes from barrier damage induced by protamine-sulfate. The same effects were seen in wild-type animals treated with the TRPC4/5 inhibitor ML204 [93]. This was also supported by in vitro data, showing an attenuation of cytoskeletal remodelling of podocytes, both after TRPC5 knockdown and pharmacological inhibition.

A role for TRPC5 in the development of focal segmental glomerusclerosis (FSGS), a leading cause of kidney failure, was also suggested by Zhou et al., who used a transgenic rat with a podocyte-specific overexpression of the angiotensin type 1 receptor (AT1R) [71]. These rats developed progressive kidney disease, and treatment of these rats with the TRPC4/5 inhibitor ML204 prevented podocyte death and attenuated the proteinuria caused by kidney damage. In addition, isolated cell studies showed increased riluzole-mediated single channel currents in rat glomeruli from AT1R animals, again inhibited by ML204. This study also introduced a novel TRPC5 inhibitor, named AC1903 (see Section 3.2.2), which showed similar effects to ML204 in suppressing proteinuria, in both AT1R transgenic animals and a model of hypertension-induced FSGS. However, a role of TRPC5 in progressive kidney disease was not supported by a study with transgenic mice overexpressing either wild-type TRPC5 or a dominant-negative TRPC5 mutant [94]. No difference in LPS-induced kidney damage was seen between the different animal groups, and treatment with the inhibitor ML204 (3 × 2 mg/kg) showed no effect on proteinuria in LPS-challenged animals. Treatment with the TRPC1/4/5 activator (−)EA (3 mg/kg, 24 h apart, i.p.) had no adverse effects on proteinuria in mice. However, as (−)EA displays poor stability in rodent plasma [51], it is debatable whether blood levels of (−)EA would reach levels sufficient to activate kidney TRPC5 channels and cause kidney damage. It is important to note, as stated by Van der Wijst and Bindels [95], that the two studies above used different doses of ML204 and different dosing regimens, which could account for the different effects seen. In addition, the exact identity of the TRPC1/4/5 channels expressed in the kidney is not known, and neither is the exact role of TRPC6 in FSGS. The channels may contain TRPC1, and although ML204 has recently been found to be a weak inhibitor of (−)EA-activated TRPC1:C5 channels, it is unknown how potent ML204 is against riluzole-activated channels. Overexpression of TRPC5 alone may not lead to formation of more channels linked to the studied phenotype, and knockout of *Trpc5* may lead to changes in formation of tetrameric ion channels by non-affected proteins such as TRPC1.

### 4.3. Roles of TRPC1/4/5 Channels in Cancer

It is well established that intracellular Ca^2+^ homeostasis is altered in cancer and that dysregulation of Ca^2+^ signalling is involved in tumour initiation, progression, metastasis, and angiogenesis. The roles of TRPC4 and TRPC5 in migration/proliferation of cancer cells, angiogenesis, cancer cell multi-drug resistance and (−)EA-induced renal cancer cell death have been reviewed in 2016 [96]. Here, we highlight the use of small-molecule TRPC1/4/5 modulators in the study of specific vulnerabilities of A498 renal carcinoma and SW982 synovial sarcoma cancer cells.

The discovery that the natural product (−)EA displays highly potent and selective cytotoxicity against eight renal cancer cell lines (GI_50_ values of under 1–87 nM; >1000-fold selectivity over other cell lines) led to target identification studies by several groups. PKCθ [56] and TRPC1:C4 channels (see Section 3.1.1) [14,15,51] have been proposed as the relevant target of (−)EA in renal cancer cells, and both proposals were based on extensive experimentation. A discussion of the evidence for these proposed mechanisms-of-action was included in a recent review on the englerins by Wu et al. [55]. It is possible that activation of TRPC1:C4 and effects on PKCθ-mediated gene regulation are linked in some cancer cells. However, in A498 cells and SW982 cells, both the potency of (−)EA in cell death assays (low nanomolar concentrations) and rapid onset of effects (minutes) are consistent with activation of TRPC1:C4 channels in these cells (low nanomolar EC_50_ values; similar to the potency in excised membrane patches from HEK293 cells containing TRPC1:C4 channels), but not with the proposed direct effects on PKCθ (majority of effects reported with 1–10 µM of (−)EA) and (much slower) downstream effects on gene transcription [14,15,16,51]. Furthermore, application of a PKCθ inhibitor has no effect on A498 cell proliferation and does not protect the cells from (−)EA-induced toxicity [51]. The analysis of >500 well characterized cancer cell lines revealed that TRPC4 mRNA abundance is the feature best correlated with sensitivity to (−)EA [51]. Knockdown of either TRPC1 or TRPC4 protects A498 and SW982 cells against (−)EA [14,15,16], while the Na^+^/K^+^ ATPase inhibitor ouabain increases the cytotoxic effect of (−)EA [15,16]. In addition, the well-characterised, highly potent and selective TRPC1/4/5 inhibitor Pico145 (see Section 3.2.1) strongly inhibits the cytotoxicity of (−)EA in SW982 cells [16], demonstrating the value of high-quality chemical probes in target validation studies. Overall, these studies suggest that (−)EA achieves its effect in A498 and SW982 cells through induction of sustained Na^+^ entry through TRPC1:C4 channels, and that expression of functional TRPC1:C4 channels is necessary for potent and rapid (−)EA-induced cytotoxicity in these cell lines. In addition, Carson et al. demonstrated that overexpression of TRPC4 is sufficient to make HEK293 cells sensitive to growth inhibition by nanomolar concentrations of (−)EA (IC_50_ = 28 nM) [51].

A recent report by Wei et al. suggests that the expression of TRPC4-containing channels in medulloblastoma cells (which also express TRPC1 and TRPC5) promotes cell migration, contributing to invasion/metastasis [97]. However, the exact composition of these channels is not known, and inhibitory effects of (−)EA on migration of these cells are difficult to correlate with TRPC1/4/5 channel activity because the high concentration of (−)EA (10 µM) used in this study may affect additional mechanisms.

### 4.4. Roles of TRPC1/4/5 Channels in the Cardiovascular System

Several members of the TRP channel family have been implicated in the cardiovascular system, including TRPC1/4/5 (for reviews, see [98,99,100,101]). Here, we highlight a few recent examples. Based on studies in mice and rats, a role of TRPC5 channels as baroreceptor mechanosensors that regulate blood pressure has been suggested [73], although there was an error in the original published data and the findings have been challenged [102,103], indicating that further studies are needed.

Camacho Londoño et al. found that a background Ca^2+^ entry pathway that fine-tunes Ca^2+^ cycling in cardiomyocytes critically depends on TRPC1 and TRPC4 proteins. Suppression of this channel activity by *Trpc1/Trpc4* double knockout protects against pathological cardiac remodelling in mice, without affecting normal cardiac function [81].

TRPC4 channels have been studied in the development of pulmonary hypertension. Alzoubi et al. reported that *Trpc4* inactivation in rats confers a survival benefit in severe pulmonary arterial hypertension [78]. This was attributed to decreased occlusive remodelling of blood vessels in knockout animals. Recent reports suggest that TRPC4 channels are implicated in (bacterial toxin-induced/aggravated) pulmonary arterial hypertension/stenosis by increasing proliferation/permeability of endothelial and smooth muscle cells [79,80,104].

To date, no pharmacological modulators of TRPC1/4/5 have been reported in models of pathological cardiac remodelling or pulmonary hypertension.

### 4.5. Additional Roles and Opportunities

*Trpc5* knockout animals have been used in the study of hepatic dyslipidaemia by Alawi et al. [105]. The authors compared cholestasis-induced liver injury in wild-type compared to *Trpc5* knockout mice, and found significantly reduced injury in knockout animals, as well as reduced dyslipidaemia and hypercholanemia, suggesting that TRPC5 channels could be involved in liver function.

TRPC5 channels have also been implicated in arthritis, although their role is unclear. Human fibroblast-like synoviocytes, the cells that secrete synovial fluid, express TRPC1 and TRPC5, and blocking their activity using antibodies or siRNA increases the secretion of matrix metalloproteases (MMPs), which is thought to lead to tissue remodelling and arthritis [34]. Additionally, TRPC5 channel expression is increased in the synovium in a mouse model of arthritis, and genetic deletion of TRPC5 as well as pharmacological inhibition with ML204 exacerbate arthritis induced by injection of complete Freud’s adjuvant [106]. Whether pharmacological activation of TRPC5 is beneficial in arthritis is as yet unknown, but these results suggest a potentially protective role for TRPC5.

Mature adipocytes in murine and human perivascular fat express TRPC1 and TRPC5, and contain constitutively active channels with an I–V profile consistent with TRPC1:C5 channels [17]. These mature adipocytes also suppress excretion of adiponectin, an adipokine known to have anti-inflammatory, anti-atherosclerotic, and insulin-sensitising effects. Inhibition of TRPC1:C5 currents by genetic methods (RNAi or over-expression of a dominant-negative TRPC5 ion pore mutant) led to increased adiponectin secretion from adipocytes. The same effect was seen in vitro when TRPC1:C5 channels were blocked with a TRPC5 antibody or dietary fatty acids, revealing a potential mechanism for cardioprotection by these fatty acids.

TRPC channels have been implicated in diabetes-associated complications [107], and a recent study suggests that Ca^2+^-permeable channels containing TRPC1 inhibit exercise-induced protection against high-fat diet-induced obesity and type II diabetes [108]. In addition, a recent study with *Trpc1/4/5/6*^−/−^ mice suggests that TRPC channels contribute to the development of diabetic retinopathy. Knockout mice were protected from hyperglycaemia-evoked vasoregression and STZ-induced thinning of the retinal layer. These effects may be due to a role of TRPC channels in the regulation of expression/activity of glyoxalase 1 (GLO1), a key enzyme involved in the detoxification of the reactive metabolite methylglyoxal [109]. The exact nature of the channels involved in this phenotype is not known, and the effect of pharmacological TRPC1/4/5 modulators has not been reported yet.

## 5. Conclusions

Small-molecule modulators of TRPC1/4/5 channels can complement genetic approaches in dissecting the different roles of specific TRPC1/4/5 channels across species, tissues, and pathologies (see Section 4). Whereas genetic perturbation of TRPC proteins (overexpression, knockout/knockdown) can be performed with high precision, it may lead to secondary effects caused by alterations in native channel stoichiometries and protein–protein interactions, complicating data interpretation (and potentially masking the full potential of the channels as therapeutic targets) [110]. In contrast, chemical probes generally act quickly, acutely, reversibly, and can be used for experiments in cells, tissues and animals [111,112,113]. High-quality chemical probes are powerful tools in target validation studies, and can serve as useful starting points for drug development; however, their development is often costly in terms of time and resources. In addition, even high-quality chemical probes are likely to have off-targets, and potency and selectivity of a chemical probe may be dependent on cellular context (expression levels and localisation of targets, post-translational modifications, protein–protein interactions, presence of endogenous modulators, etc.). For these reasons, functional studies that carefully combine genetic and pharmacological approaches—and take limitations of both into account—are highly recommended for unravelling the biological roles of specific TRPC1/4/5 channels.

Traditionally, small-molecule modulation of TRPC1/4/5 channels has often relied on small-molecule modulators with low potency/selectivity. The emergence of highly potent and highly selective TRPC1/4/5 modulators such as (−)EA, Pico145 and HC-070 now offers unprecedented opportunities for TRPC1/4/5 research, and especially Pico145 and HC-070 are suitable for in vivo studies. The toxicity (depending on administration mode) [51,55,60] and instability of (−)EA in plasma and the digestive system [51,60] may limit its use for such studies though, and other activators such as BTD and riluzole have to be used at micromolar concentrations, increasing the chance of off-target effects. Therefore, additional potent and selective TRPC1/4/5 activators are needed. 

The differentiation between different TRPC1/4/5 tetramers by compounds such as Pico145, ML204 and AC1903 suggests that development of tetramer-specific modulators is possible, and structural studies of TRPC1/4/5 channels may inform such developments. Such modulators could be used to reveal the composition of TRPC1/4/5 channels implicated in different (patho)physiological processes, which so far is often poorly understood (see Section 4). Studies of the roles of TRPC1/4/5 channels in anxiety, (−)EA-mediated cancer cell death and progressive kidney disease highlight the necessity and usefulness of small-molecule TRPC1/4/5 modulators for biological research. In addition, these studies show that it is essential to use carefully selected chemical probes and control compounds (with different selectivity profiles), at concentrations that are sufficient (and not much higher than that) to modulate the relevant TRPC1/4/5 channels under the tested conditions, and—where possible—to test dose-dependence of effects. When used appropriately, TRPC1/4/5 modulators can transform the understanding of TRPC1/4/5 channels in health and disease, and of the advantages and disadvantages of TRPC1/4/5 channels as drug targets.

## Figures and Tables

**Table 1:** Overview of selected TRPC1/4/5 activators.

Compound Name	Targets (EC_50_)	Potential Off-Targets	Comments	References
(−)-englerin A	TRPC1/4/5 (1–10 nM)	PKCθ, Ca_V_1.2 (µM concentrations needed)	High selectivity and efficacy; unstable in rodent plasma/GI tract	[14,15,51,56,57]
BTD	TRPC5:C5 (1.3 µM) TRPC1:C5, TRPC4:C5	TRPM8 (EC_50_ = 20.6 µM)	No effect on TRPC4:C4, TRPC1:C4, or other tested TRP channels	[61]
Riluzole	TRPC5:C5 (9.2 µM), TRPC1:C5	Multiple ion channels	Marketed drug	[64,65]

**Table 2:** Overview of selected TRPC1/4/5 inhibitors.

Compound Name	Targets (IC_50_; Activator)	Potential Off-Targets	Comments	References
Pico145/HC-608	TRPC1/4/5 (0.03–1.3 nM; (−)EA)	Not known	Highly selective; suitable for in vivo use	[19,66,67]
HC-070	TRPC1/4/5 (0.3–2 nM; La^3+^ or carbachol/muscarinic receptors)	Not known	Highly selective; suitable for in vivo use	[66,67]
AC1903	TRPC5:C5 (14.7 µM)	Not known	No known effect on TRPC4:C4, TRPC6:C6 and kinases; suitable for in vivo use	[71]
